# Outcome‐specific risk factors for dehydration and acute kidney injury readmission after ileostomy: A US National Cohort Study, 2016–2022

**DOI:** 10.1111/codi.70656

**Published:** 2026-10-26

**Authors:** Anmol Nigam, Chidiebere Onongaya, Pravin Meshram, James V. Harmon

**Affiliations:** ^1^ Department of Surgery University of Minnesota Minneapolis Minnesota USA

**Keywords:** acute kidney injury, dehydration, ileostomy, patient readmission, risk factors

## Abstract

**Aim:**

Single‐centre pathways have reduced dehydration and acute kidney injury (AKI)‐specific readmission after ileostomy from 17%–28% to single‐digit rates, but whether these gains translated nationally is unknown. We examined how discharge destination relates to outcome‐specific readmission, quantified 30‐day readmission rates and encounter costs from 2016 to 2022 and identified risk factors at the population level.

**Method:**

Retrospective cohort study using the United States Nationwide Readmissions Database (NRD, 2016–2022). Adults undergoing ileostomy creation were included. The primary outcome was 30‐day readmission for dehydration or AKI. Survey‐weighted multivariable logistic regression identified risk factors for composite readmission, with home‐discharge effects compared between dehydration‐ and AKI‐specific outcomes. Inflation‐adjusted encounter costs were calculated.

**Results:**

Among 189,829 patients (176,150 discharged alive), 24,024 were readmitted within 30 days (13.5%, 95% CI 13.3–13.7). Home discharge had opposing associations by outcome subtype. Dehydration‐specific readmission increased (aOR 1.25) and AKI‐specific readmission decreased (aOR 0.92, *p* < 0.001 for interaction). The strongest risk factors were extended length of stay >7 days (aOR 1.48), complicated diabetes (aOR 1.46) and age ≥ 65 years (aOR 1.46). Annual rates were stable (*p* for trend = 0.826). Readmission encounters totalled $1.51 billion in inflation‐adjusted dollars. The principal‐diagnosis lower‐bound estimate was $0.40 billion.

**Conclusion:**

Home discharge had divergent associations by outcome subtype, identifying the post‐discharge transition as a candidate for prospective intervention testing. Stagnant national rates over 7 years despite established prevention evidence, an income‐dependent gradient and a graded rate across cumulative risk‐factor burden together motivate prospective evaluation of risk‐stratified discharge protocols incorporating community stoma‐output monitoring.

**What does this paper add to the literature?**

In a US national cohort, home discharge after ileostomy showed opposing associations with dehydration‐ versus AKI‐specific readmission (aOR 1.25 vs. 0.92, interaction *p* < 0.001), supporting the post‐discharge transition as a candidate for prospective intervention testing. National 30‐day rates remained stable from 2016 to 2022 (13.5%, *p*‐trend 0.826) despite published prevention work.

## INTRODUCTION

US single‐centre cohorts have reported 17% 30‐day dehydration or renal failure readmission and 16.9%–28% 60‐day all‐cause readmission [1, 2, 3]. International systematic reviews estimate 5%–6% 30‐day dehydration‐specific readmission, while a Japanese multicentre cohort reported 22.4% ileostomy‐associated kidney injury using creatinine‐based Kidney Disease Improving Global Outcomes criteria before stoma closure [4, 5, 6]. These estimates are not directly comparable because their outcome definitions and follow‐up periods differ. High‐output stomas produce fluid and electrolyte losses that drive this morbidity [7, 8, 9], yet whether published prevention work over the past decade has moved US national readmission rates remains untested.

A prior US national database study examined all‐cause readmissions after ileostomy creation in the International Classification of Diseases (ICD)‐9 era [10], but did not characterize dehydration‐ or acute kidney injury (AKI)‐specific outcomes, national temporal trends or encounter costs. Prior single‐centre and National Surgical Quality Improvement Program (NSQIP) work has produced a weighted prediction score (DRIP, AUC 0.71–0.74) [11], a dehydration risk calculator [12], and a multidisciplinary programme reducing dehydration to 4.4% [13], but cannot track cross‐institution readmissions.

We used the Nationwide Readmissions Database to examine how discharge destination relates to outcome‐specific readmission for dehydration or AKI after ileostomy creation, quantify national 30‐day rates and encounter costs over time and identify risk factors at population scale. We hypothesized that discharge destination would be differentially associated with dehydration‐ versus AKI‐specific readmission, and that identifiable patient and surgical factors would be independently associated with composite readmission with a graded rate across cumulative risk‐factor burden.

## METHOD

### Data source

We conducted a retrospective cohort study using the United States Nationwide Readmissions Database (NRD) for years 2016 through 2022. The study period covered 2016 (first full ICD‐10 year) through 2022 (most recent NRD release). The NRD contains approximately 16.5 million unweighted discharges annually from 30 participating states, accounting for approximately 60% of US hospitalizations [14]. Survey weights provided by HCUP are applied to these records to generate nationally representative estimates. Verified patient linkage permits readmissions to be tracked within each calendar year. This study was considered exempt from institutional review board review because it used de‐identified administrative data, and informed consent was waived for the same reason.

### Study population

The cohort included adults ≥18 years who underwent ileostomy creation, identified by ICD‐10 procedure codes (Table S2). The first qualifying admission served as the index hospitalization. Records missing key readmission‐linkage identifiers were excluded. Patients who died during the index hospitalization were retained in the full cohort but excluded from readmission analyses. No formal sample size calculation was performed.

### Outcome definitions

The primary outcome was 30‐day composite readmission, defined via verified patient linkage as any subsequent admission within 30 days of index discharge with dehydration or AKI in any diagnosis position, as these conditions commonly appear as secondary diagnoses precipitating admission for related sequelae missed by principal‐diagnosis‐only capture. Same‐day acute‐care transfers were defined as discharge from one hospital and admission to another on the same day, with both records indicating transfer and were excluded from readmission counts. Dehydration and AKI were defined using ICD‐10 diagnosis codes E86.x and N17.x, respectively (Table S2). Patients meeting both definitions were counted once in the composite outcome and included in both condition‐specific analyses. Each patient was counted once per readmission outcome regardless of the number of qualifying admissions. Time‐to‐event analyses used the first qualifying readmission. Secondary outcomes included 60‐day and 90‐day composite, dehydration‐specific, AKI‐specific and all‐cause readmission. Condition‐specific analyses are reported separately (Table S1).

### Predictor variables

Patient factors included age 65 years or older (defined by Medicare eligibility), sex and selected Elixhauser comorbidities [15, 16, 17] identified from index‐admission diagnosis codes. Model predictors were selected based on their clinical relevance to fluid balance, renal vulnerability and readmission after ileostomy. The selected Elixhauser conditions were renal failure, complicated diabetes, complicated hypertension, congestive heart failure and fluid and electrolyte disorders. The Elixhauser renal failure category included chronic and unspecified kidney failure, hypertensive kidney disease and renal transplant or dialysis status, but excluded N17.x codes for AKI and was not restricted to end‐stage kidney disease or dialysis. Diabetes and hypertension were categorized as complicated or uncomplicated according to the mutually exclusive Quan‐Elixhauser definitions [16]. Median household income quartile for patient ZIP code served as a socioeconomic proxy.

Surgical variables included admission urgency, underlying indication and concurrent colorectal resection. Underlying indication was classified as colorectal cancer, IBD, diverticular disease or Other using diagnosis codes listed in Table S2. Other was a residual category comprising patients without any of these diagnosis codes. The 0D1B procedure prefix identified ileostomy creation. Concurrent colorectal resection was identified using the specified procedure codes listed in Table S2. Cases were compared according to the presence or absence of concurrent colorectal resection.

*Discharge‐related factors* included discharge destination (home vs. facility) and extended length of stay (>7 days), a threshold previously associated with elevated readmission risk after colorectal surgery [18], corresponding to the 75th percentile among patients without the coded postoperative complications listed in Table S2. NRD does not separately encode postoperative length of stay, so total admission length is used. Hospital factors included bed size and teaching status.

### Statistical analysis

Continuous variables were reported as medians with interquartile ranges. Patient counts are reported unweighted unless specified otherwise. Rates, percentages and regression estimates were calculated using the NRD survey design.

All analyses incorporated the NRD complex survey design, including discharge‐level weights, hospital‐level clustering and sampling stratification, to produce nationally representative estimates. Cumulative incidence of readmission was estimated using the Kaplan–Meier method, plotted as one minus the survival function. Time‐to‐event analyses used administrative censoring at the NRD calendar‐year boundary. Fixed‐time regression analyses excluded patients whose outcome status remained indeterminate before the corresponding time point without a preceding readmission. Multivariable logistic regression identified factors associated with 30‐day readmission, with adjusted odds ratios and 95% confidence intervals. All predictors were entered simultaneously into each model. No univariable screening or automated variable‐selection procedure was used. Model convergence was confirmed for all fitted models. Benjamini‐Hochberg correction for the false discovery rate was applied across each predictor set, and all predictors significant at *p* < 0.05 remained significant after correction. Model discrimination was assessed via the C‐statistic. Temporal trends were assessed using logistic regression treating year as continuous. Subgroup analyses were performed by indication, with versus without concurrent colorectal resection, admission urgency, age and index‐admission renal failure. A sensitivity analysis restricted the outcome to principal diagnosis position (Table S3). Subtype‐specific estimates were obtained from separately fit models. The home‐discharge coefficient was formally compared using a Wald test on the coefficient difference, with the test conservative under expected positive correlation between the two outcomes. No model predictors had missing data. Total charges, used only in the cost analyses, were missing for 0.91% of patients and were excluded from cost calculations.

To summarize cumulative risk burden, patients were stratified by the count of independent risk‐increasing predictors identified in the multivariable model (elective admission, complicated diabetes, fluid/electrolyte disorders, complicated hypertension, renal failure, congestive heart failure, female sex, extended length of stay >7 days and age ≥ 65). Tiers were defined using fixed integer‐count boundaries for clinical interpretability as Low (0–1), Medium (2), High (3–4) and Very High (≥5). A linear trend in readmission rate across tiers was assessed.

Hospital costs were derived from total charges using hospital‐specific cost‐to‐charge ratios from Centres for Medicare and Medicaid Services cost reports. For multi‐year analysis, costs were adjusted for inflation using the Bureau of Labor Statistics Hospital Services Consumer Price Index and expressed in 2022 US dollars. For patients with multiple readmissions, costs were summed across all readmission encounters within 30 days. Between‐group cost comparisons used rank‐based testing.

Analyses were performed using Python 3.13 and R 4.4. Statistical significance was set at a two‐tailed alpha of 0.05. This study adhered to the Strengthening the Reporting of Observational Studies in Epidemiology (STROBE) guidelines [19].

## RESULTS

Among 207,341 ileostomy discharges, 5,981 were excluded because patients were younger than 18 years. Additional data‐quality exclusions are reported as a combined category in Figure S1 due to HCUP small‐cell reporting restrictions. After exclusions, the cohort comprised 189,829 unweighted patients (representing an estimated 350,805 weighted hospitalizations nationally, Figure S1, Table 1). The median age was 61 years (IQR 50–71), and 49.2% were female. The most common indications were colorectal cancer (31.0%), IBD (16.3%) and diverticulitis (14.3%). Diagnoses documented within the Other indication category are reported in Table S6. Ileostomy with concurrent colorectal resection occurred in 53.7% and without concurrent resection in 46.3%. Elective admission accounted for 48.6% of cases. A total of 13,679 patients (7.1%) died during index hospitalization. In‐hospital mortality ranged from 0.7% among elective IBD admissions to 17.3% among emergent admissions in the Other category (Table S4). Subsequent analyses are restricted to the 176,150 patients discharged alive. The 30‐day composite readmission rate was 13.5% (95% confidence interval [CI] 13.3%–13.7%, *n* = 24,024). Of these 24,024 patients, 5,704 met the dehydration definition only, 6,007 met the AKI definition only, and 12,313 met both definitions, representing 51.3% of the unweighted composite count. The 30‐day composite outcome was classifiable for all 176,150 patients. Composite readmission increased to 17.4% (95% CI 17.2%–17.7%) at 60 days and 19.6% (95% CI 19.4%–19.9%) at 90 days (Figure 1). The 60‐ and 90‐day regression analyses included 171,810 and 167,823 patients, respectively, excluding 4,340 and 8,327 patients censored before the corresponding time point without an observed readmission. Annual rates remained stable from 13.2% to 14.1% across 2016–2022 (*p* for trend = 0.826, Figure 2 and Table S5).

**TABLE 1:** Baseline characteristics of patients undergoing ileostomy creation.

Variable	Level	*n*	%	Median (IQR)
Age (years)	Continuous	–	–	61 (50–71)
Sex	Male	95,841	50.8%	–
	Female	93,988	49.2%	–
Primary insurance	Medicare	84,559	44.2%	–
	Medicaid	24,285	12.4%	–
	Private	72,068	38.5%	–
	Self‐pay/Other	8,917	4.9%	–
Median household income quartile	Q1 (Lowest)	47,704	26.6%	–
	Q2	48,986	27.1%	–
	Q3	48,583	25.2%	–
	Q4 (Highest)	44,556	21.2%	–
Indication for ileostomy	Colorectal Cancer	59,258	31.0%	–
	IBD	30,573	16.3%	–
	Diverticulitis	27,398	14.3%	–
	Other	72,600	38.4%	–
Concurrent colorectal resection	Present	101,209	53.7%	–
	Absent	88,620	46.3%	–
Elective admission	Yes	91,583	48.6%	–
	No	98,246	51.4%	–
*Comorbidities*				
Diabetes (complicated)	Yes	18,696	10.0%	–
Diabetes (uncomplicated)	Yes	15,765	8.3%	–
Fluid and electrolyte disorders	Yes	84,466	44.9%	–
Renal failure	Yes	21,092	11.3%	–
Congestive heart failure	Yes	19,325	10.2%	–
Hypertension (complicated)	Yes	25,746	13.7%	–
Hypertension (uncomplicated)	Yes	64,999	34.4%	–
Obesity	Yes	29,973	16.1%	–
Metastatic cancer	Yes	30,375	15.7%	–
Weight loss	Yes	49,749	26.1%	–
*Hospital characteristics*				
Hospital bed size	Small	24,129	13.1%	–
	Medium	47,435	24.4%	–
	Large	118,265	62.4%	–
Hospital teaching status	Urban teaching	153,783	81.5%	–
	Urban non‐teaching	28,386	13.7%	–
	Rural	7,660	4.8%	–

*Note*: *n* represents unweighted sample counts. Percentages are survey‐weighted estimates of the national population. Other indicates the absence of diagnosis codes for colorectal cancer, IBD or diverticular disease. Hospital bed‐size categories follow HCUP definitions.

Abbreviations: IBD, inflammatory bowel disease; IQR, interquartile range.

**FIGURE 1:**
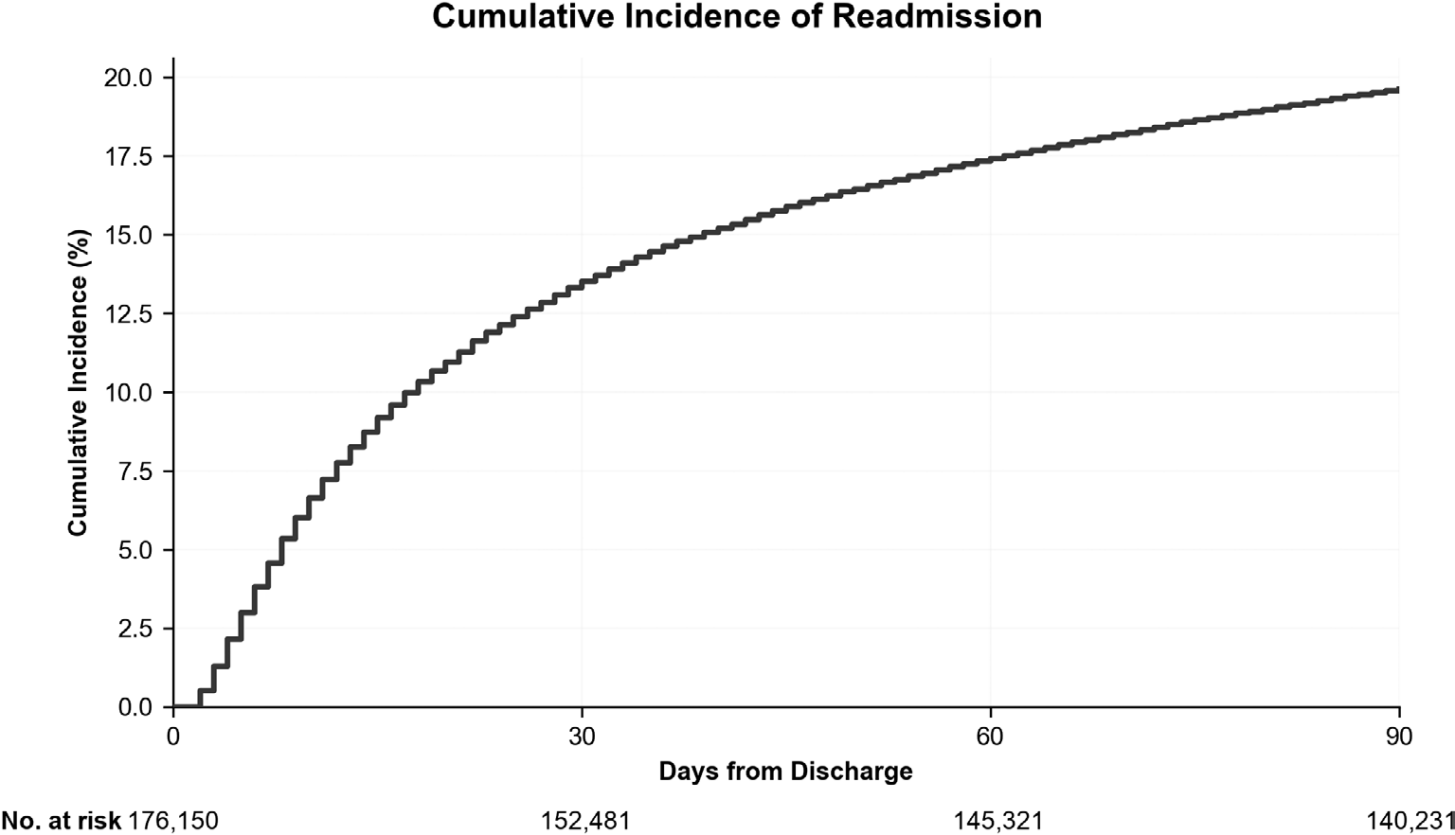
Cumulative incidence of readmission for dehydration or acute kidney injury after ileostomy creation. Patients administratively censored at NRD calendar‐year boundary. NRD, Nationwide Readmissions Database.

**FIGURE 2:**
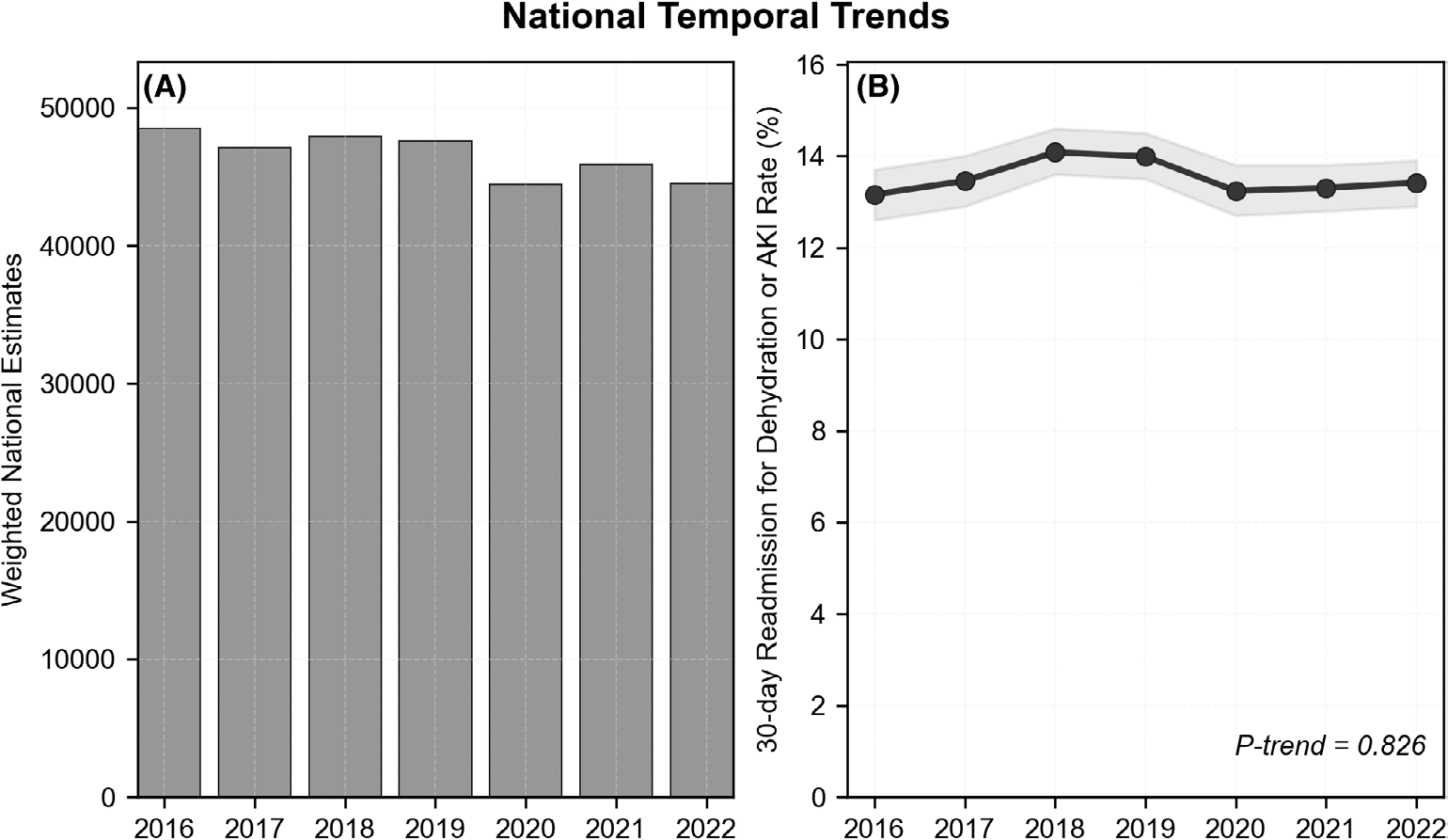
National temporal trends in 30‐day readmission for dehydration or acute kidney injury after ileostomy creation, 2016 to 2022. (A) Survey‐weighted national estimates of ileostomy creation discharges per year. (B) Annual 30‐day readmission rate for dehydration or acute kidney injury (point estimates with 95% confidence intervals). P‐trend from survey‐weighted logistic regression with year as a continuous predictor. Exact annual estimates are reported in Table S5.

On multivariable logistic regression, the strongest predictors of 30‐day readmission were extended length of stay greater than 7 days (adjusted odds ratio [aOR] 1.48, 95% CI 1.42–1.53), complicated diabetes (aOR 1.46, 95% CI 1.39–1.53), and age 65 years or older (aOR 1.46, 95% CI 1.41–1.51, all *p* < 0.001, Table 2, Figure 3). Additional significant predictors included fluid and electrolyte disorders, renal failure, complicated hypertension, lowest income quartile, elective admission and female sex (aORs 1.08–1.36, all *p* < 0.001), as well as congestive heart failure (aOR 1.07, *p* = 0.03). Ileostomy with concurrent colorectal resection (aOR 0.89, 95% CI 0.86–0.92) and IBD (aOR 0.92, 95% CI 0.87–0.96) were associated with lower readmission risk (both *p* < 0.001). In stratified analysis, the IBD effect was concentrated in AKI‐specific readmission (aOR 0.74, 95% CI 0.70–0.78) with no significant association with dehydration‐specific readmission (aOR 0.99, 95% CI 0.94–1.05, Table S1). The model C‐statistic was 0.639 (95% CI 0.635–0.644). In the principal‐diagnosis sensitivity analysis (4.8%, *n* = 8,533), extended length of stay, complicated diabetes and age ≥ 65 remained the strongest predictors and retained the same direction of association (aOR 1.49, 1.45 and 1.84, respectively, Table S3).

**TABLE 2:** Risk factors for 30‐day readmission for dehydration or acute kidney injury.

Risk factor	aOR (95% CI)	*p*‐value
Discharged home (vs. Facility)	1.00 (0.96–1.05)	0.854
Colorectal cancer (vs. No)	0.99 (0.96–1.03)	0.655
Diverticular disease (vs. No)	0.98 (0.94–1.02)	0.264
Inflammatory bowel disease (vs. No)	0.92 (0.87–0.96)	<0.001
Elective admission (vs. No)	1.09 (1.05–1.13)	<0.001
Congestive heart failure (vs. No)	1.07 (1.01–1.13)	0.034
Diabetes (complicated) (vs. No)	1.46 (1.39–1.53)	<0.001
Fluid/Electrolyte disorder (vs. No)	1.36 (1.31–1.41)	<0.001
Hypertension (complicated) (vs. No)	1.18 (1.09–1.26)	<0.001
Renal failure (vs. No)	1.28 (1.20–1.37)	<0.001
Sex (vs. Male)	1.08 (1.05–1.12)	<0.001
Hospital bed size (Large)	Ref	–
Hospital bed size (Medium)	1.05 (1.01–1.09)	0.020
Hospital bed size (Small)	1.03 (0.98–1.08)	0.218
Urban/rural teaching (Urban teaching)	Ref	–
Urban/rural teaching (Rural)	0.97 (0.90–1.05)	0.476
Urban/rural teaching (Urban non‐teaching)	1.05 (1.01–1.09)	0.028
Extended length of stay (>7 days) (vs. No)	1.48 (1.42–1.53)	<0.001
Household income (Q4 (Highest))	Ref	–
Household income (Q1 (Lowest))	1.17 (1.12–1.23)	<0.001
Household income (Q2)	1.13 (1.08–1.18)	<0.001
Household income (Q3)	1.03 (0.99–1.08)	0.178
Age ≥ 65 years (vs. No)	1.46 (1.41–1.51)	<0.001
Ileostomy with concurrent colorectal resection (vs. without)	0.89 (0.86–0.92)	<0.001

*Note*: *n* = 176,150 patients discharged alive; 24,024 events. C‐statistic = 0.639 (95% CI 0.635–0.644). See Table S1 for outcome‐specific results (dehydration‐specific, AKI‐specific, 60‐day, 90‐day).

Abbreviations: aOR, adjusted odds ratio; CI, confidence interval; Ref, reference category.

**FIGURE 3:**
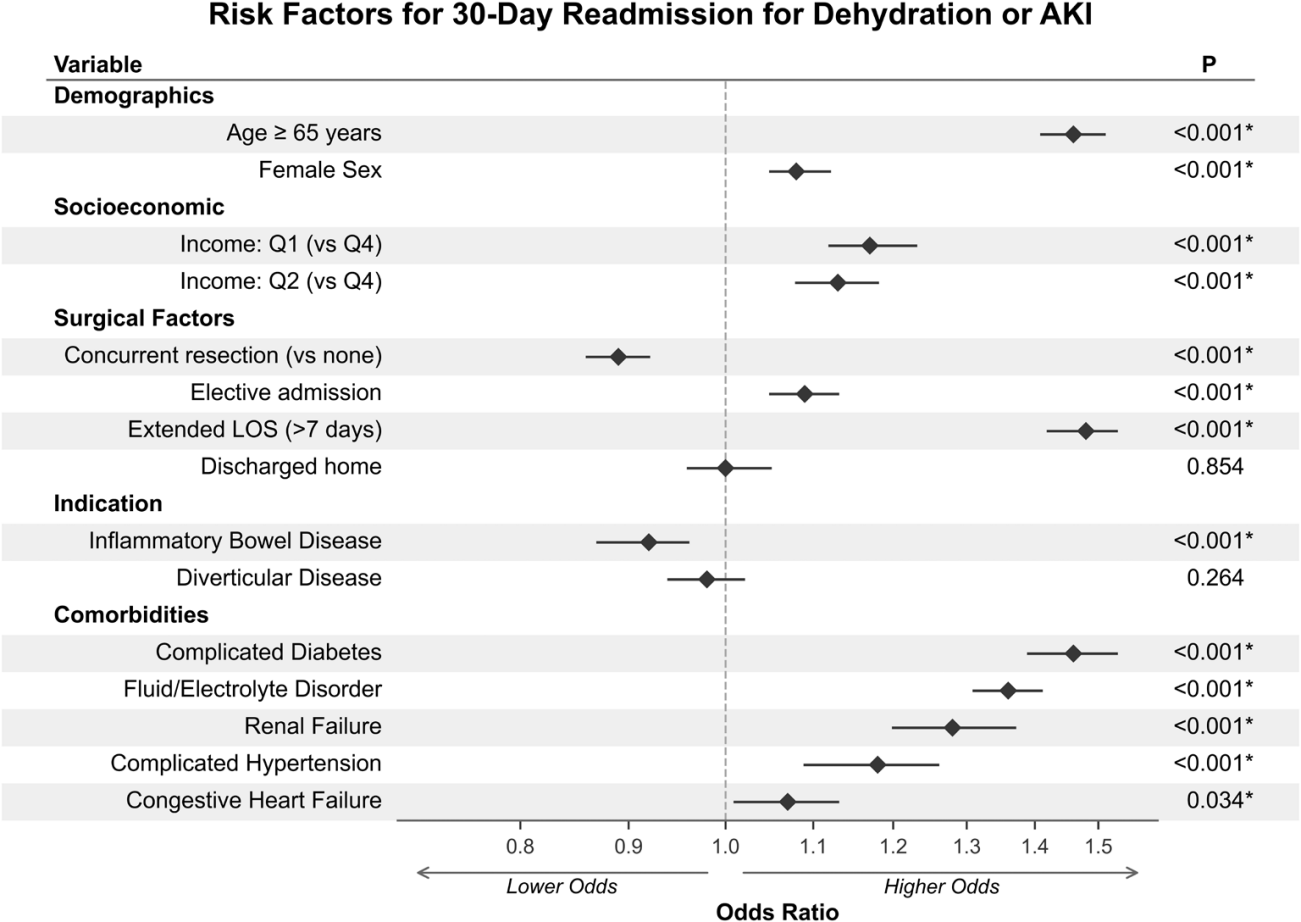
Risk factors for 30‐day readmission for dehydration or acute kidney injury. Adjusted odds ratios with 95% confidence intervals from survey‐weighted multivariable logistic regression (all predictors entered simultaneously). Dashed vertical line indicates null (OR = 1.0). *p < 0.05. aOR, adjusted odds ratio; LOS, length of stay; Q1–Q4, household income quartiles.

Discharge destination showed divergent associations by outcome subtype. Home discharge was not associated with composite readmission (aOR 1.00, 95% CI 0.96–1.05, *p* = 0.854). In stratified analysis, home discharge was associated with increased dehydration‐specific readmission (aOR 1.25, 95% CI 1.19–1.31) and decreased AKI‐specific readmission (aOR 0.92, 95% CI 0.88–0.96, Figure 4, Table S1). The home‐discharge coefficient differed significantly between the two models (p for interaction < 0.001, OR ratio [dehydration vs. AKI] = 1.36, 95% CI 1.27–1.45).

**FIGURE 4:**
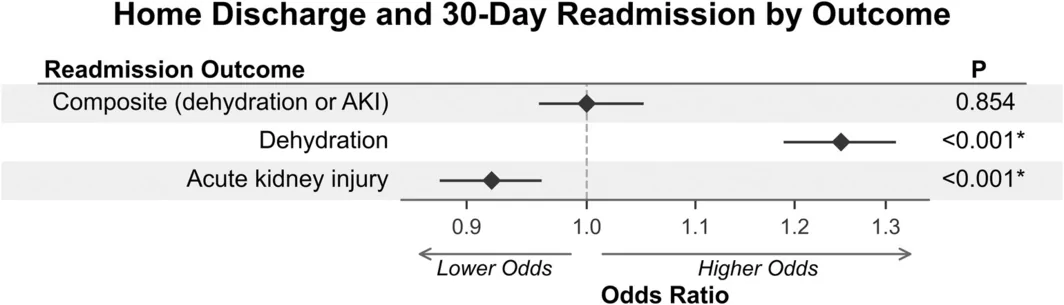
Differential effect of discharge to home on 30‐day readmission outcome type. Adjusted odds ratios with 95% confidence intervals from survey‐weighted multivariable logistic regression of three separate outcomes: Composite readmission (dehydration or acute kidney injury), dehydration‐specific readmission and acute kidney injury‐specific readmission. Reference line indicates null (OR = 1.0). All models adjusted for the same predictor set as Table 2. *p < 0.05; n.s., not significant. Source data in Table S1.

Readmission rates increased with cumulative risk‐factor burden, from 7.1% (95% CI 6.8%–7.5%) in the Low tier (0–1 factors) to 9.7% (95% CI 9.4%–9.9%) at Medium (2), 14.7% (95% CI 14.4%–15.0%) at High (3–4) and 19.5% (95% CI 19.0%–20.0%) at Very High (≥5, *p* for trend <0.001). Subgroup rates were concordant with the gradient. The readmission rate was 23.2% among patients with index‐admission renal failure versus 12.4% among those without, 17.7% among patients aged 65 years or older versus 10.9% among younger patients, and 15.4% after non‐elective versus 11.7% after elective admission (all unadjusted).

Among all‐cause readmission encounters, the leading principal diagnoses were aftercare (21.0%), acute and unspecified renal failure (12.1%), septicemia (8.9%), postoperative digestive complications (8.9%) and fluid and electrolyte disorders (4.4%). Median index admission cost was $34,616 (IQR $22,806–$58,564). Patients readmitted within 30 days for dehydration or AKI had higher index costs (median $40,480, IQR $26,165–$69,698) than those not readmitted ($33,821, IQR $22,368–$56,838, *p* < 0.001). The national index admission burden across the cohort was $20.26 billion over the study period, of which $2.76 billion was incurred by the subset of patients who were subsequently readmitted within 30 days for dehydration or AKI. The readmission encounters themselves accounted for an additional $1.51 billion in survey‐weighted cost (any‐position dehydration or AKI coding). Restricting to encounters with dehydration or AKI as the principal diagnosis yields a lower‐bound attributable cost of $0.40 billion (26.7% of any‐position encounters).

## DISCUSSION AND CONCLUSIONS

In a US national cohort, home discharge after ileostomy showed opposing associations with dehydration‐ and AKI‐specific readmission (OR ratio 1.36, 95% CI 1.27–1.45). This outcome‐specific divergence has not been characterized in the existing literature and points to the post‐discharge transition as a candidate for prospective intervention testing. National 30‐day rates were stable across the 7‐year study period, with substantial readmission encounter cost burden in addition to index admission costs already incurred by patients later readmitted. Unchanged national outcomes despite a decade of single‐centre reports showing that targeted interventions can reduce these readmissions [13, 20] identify an implementation gap that risk factor identification alone has not closed.

Our 13.5% US national rate lies between pooled international estimates of 5%–6% for 30‐day dehydration‐specific readmission and a US single‐centre estimate of 17% for 30‐day dehydration or renal failure readmission [1, 4, 5]. Direct comparison is limited by differing outcome definitions and follow‐up intervals. Three of the four strongest predictors were extended length of stay, complicated diabetes and age 65 years or older. These describe patients with diminished physiological reserve, consistent with prior work [12, 21, 22, 23] and persistent under principal‐diagnosis‐only outcome capture (Table S3). Fluid and electrolyte disorders and renal failure documented during the index admission identified patients with greater physiological vulnerability [24]. Pencovich et al. similarly identified elevated postoperative creatinine during the index admission as an independent predictor of subsequent AKI‐related readmission after ileostomy creation [25]. Long‐term renal sequelae have been documented in defunctioning ileostomy cohorts, including post‐reversal glomerular filtration rate decline [26] and elevated AKI risk in Swedish national data [27]. Advanced age compounds these effects through declining renal function and blunted thirst response [22, 23].

Prior studies have associated angiotensin‐converting enzyme (ACE) inhibitor and angiotensin receptor blocker (ARB) use with ileostomy‐related dehydration, which may partly explain the associations observed among patients with complicated diabetes and congestive heart failure [28, 29]. Pencovich et al. found diabetes associated with earlier readmission timing but not independently associated with readmission in multivariable analysis [25]. The association between congestive heart failure and readmission strengthened at later windows, but the mechanism underlying this pattern could not be determined (Table S1).

Readmission varied by underlying indication and operative context. The lower AKI‐related readmission associated with inflammatory bowel disease persisted after adjustment for age and selected comorbidities, suggesting that condition‐specific factors may contribute to this association (Table S1). ICD‐10 codes do not directly distinguish loop from end ileostomy configuration, although prior National Inpatient Sample studies have interpreted combinations of ileostomy, colorectal resection and indication‐specific codes as suggestive of operative strategy [30, 31]. The lower readmission associated with concurrent colorectal resection may therefore partly reflect differences in ileostomy configuration. Elective admission was associated with higher adjusted readmission, supporting continued attention to hydration and renal monitoring after planned ileostomy creation. Extended length of stay was also associated with readmission, consistent with nationwide colorectal data [18]. This contrasts with ileostomy‐specific studies in which shorter stays were associated with greater risk [3, 11].

Prior nationwide work has reported facility discharge as a general risk factor for readmission after surgery [32]. In this study, however, home discharge showed opposing associations with dehydration‐ and AKI‐specific readmission. These associations may reflect differences in post‐discharge monitoring and recognition. Facility‐based care may facilitate laboratory detection of AKI, whereas home management may depend more heavily on recognizing high stoma output and clinical volume depletion. Professional ostomy‐care organizations recommend structured stoma nurse follow‐up after discharge [33, 34]. A US patient and clinician survey documented inconsistent delivery of recommended ostomy‐care standards [35]. Coordinated hospital‐level collaboration has accelerated surgical quality improvement in other domains [36], offering a potential model for implementing these recommendations.

Patients in the lowest income quartile also faced significantly increased readmission, consistent with prior work in surgical populations [37, 38]. Plausible mechanisms include differential access to outpatient follow‐up, medication continuity and community stoma nursing support. Risk‐stratified discharge protocols may benefit from accounting for this gradient to ensure equitable delivery of post‐discharge care.

The cumulative risk burden gradient summarizes population‐level risk concentration at discharge. Complementary patient‐level prediction tools include the DRIP score and the Alqahtani calculator [11, 12]. Existing single‐centre interventions show what is achievable once the at‐risk population is identified. Nagle et al. reduced dehydration readmissions from 15.5% to 0% through an ileostomy pathway with early post‐discharge follow‐up [20], and Kong et al. reduced them to 4.4% with multidisciplinary monitoring [13]. Discharge medication reconciliation around ACE inhibitors, ARBs and diuretics warrants prospective evaluation, given a 13.56‐fold increase in dehydration readmission associated with these agents in a single‐centre ileostomy cohort [28] and a similar association reported in a Swedish national registry [29].

This study has limitations inherent to retrospective administrative data. The NRD does not include medication use, laboratory values, vital signs or race and ethnicity data. Calendar‐year linkage cannot capture readmissions crossing year boundaries or occurring out of state. Calendar‐year censoring also resulted in relative underrepresentation of late‐year discharges in the 60‐ and 90‐day analyses, which could affect estimates if readmission rates varied seasonally. Prospective studies should evaluate whether monitoring practices and admission thresholds across discharge settings contribute to these outcome‐specific patterns and whether targeted home stoma‐output monitoring reduces readmission.

In a US national cohort, home discharge showed opposing associations with dehydration‐ versus AKI‐specific readmission after ileostomy, identifying the post‐discharge transition, particularly home stoma‐output monitoring, as a candidate for prospective intervention testing. Despite a decade of published prevention work, national readmission rates were unchanged, with persistent encounter cost burden.

## AUTHOR CONTRIBUTIONS

**Anmol Nigam:** Conceptualization; investigation; methodology; validation; writing – review and editing; data curation; writing – original draft; visualization; formal analysis. **Chidiebere Onongaya:** Methodology; validation; writing – review and editing. **Pravin Meshram:** Methodology; validation; writing – review and editing. **James V. Harmon Jr:** Methodology; writing – review and editing; conceptualization; resources; supervision.

## FUNDING INFORMATION

This research was supported by the William W. Harmon Surgery Education and Research Fund, independent of all study decisions.

## CONFLICT OF INTEREST STATEMENT

The authors declare no conflicts of interest.

## ETHICS APPROVAL STATEMENT

This study was exempt from institutional review board approval because it used de‐identified administrative data from the Healthcare Cost and Utilization Project, Agency for Healthcare Research and Quality.

## PATIENT CONSENT STATEMENT

Informed consent was waived because the study used de‐identified administrative data.

## Supporting information


**Supplemental Figure S1.** Cohort selection flow diagram. Data‐quality exclusions are presented as a combined category due to HCUP small‐cell reporting restrictions. No admissions were excluded solely because they occurred in December. Subsequent same‐day transfers were excluded from readmission counts rather than from the patient cohort. In‐hospital deaths (*n* = 13,679) were excluded from readmission analysis. Counts are unweighted unless otherwise specified. NRD, Nationwide Readmissions Database.

**Supplemental Table S1.** Risk factors across readmission outcomes. Each column presents a complete multivariable model containing all predictors shown. Sample sizes: 30‐day composite, 176,150 (24,024 events); 30‐day dehydration, 175,986 (18,017 events); 30‐day AKI, 176,065 (18,320 events); 60‐day composite, 171,810 (30,964 events); 90‐day composite, 167,823 (34,837 events). The dehydration model excluded 164 patients with documented AKI who died in hospital before day 30 without documented dehydration. The AKI model excluded 85 patients with documented dehydration who died in hospital before day 30 without documented AKI. Both groups remained composite events. C‐statistics (95% CIs): 30‐day composite, 0.639 (0.635–0.644); 30‐day dehydration, 0.613 (0.608–0.618); 30‐day AKI, 0.675 (0.670–0.680); 60‐day composite, 0.652 (0.648–0.656); 90‐day composite, 0.655 (0.652–0.659). *p < 0.05. AKI, acute kidney injury; CI, confidence interval; aOR, adjusted odds ratio; Ref, reference category.

**Supplemental Table S2.** Procedure and diagnosis codes for cohort identification, outcome ascertainment and postoperative complications. Asterisk (*) indicates all codes beginning with that prefix were included. ICD, International Classification of Diseases.

**Supplemental Table S3.** Risk factors for 30‐day readmission for dehydration or AKI by outcome diagnosis position. Sample sizes: any position, 176,150 (24,024 events, 13.5%); principal diagnosis only, 176,106 (8,533 events, 4.8%). C‐statistics (95% CIs): any position, 0.639 (0.635–0.644); principal diagnosis only, 0.649 (0.643–0.656). *p < 0.05. AKI, acute kidney injury; CI, confidence interval; DX, diagnosis; aOR, adjusted odds ratio; Ref, reference category.

**Supplemental Table S4.** In‐hospital mortality during index admission, stratified by indication and admission type. Counts shown as unweighted sample sizes. Weighted counts are national survey‐weighted estimates using NRD discharge weights. Mortality calculated from weighted counts. IBD, inflammatory bowel disease.

**Supplemental Table S5.** Annual 30‐day readmission rates for dehydration or acute kidney injury after ileostomy creation. The trend‐test *p*‐value was 0.826. CI, confidence interval.

**Supplemental Table S6.** Diagnoses documented among patients in the Other indication category. The Other indication category was defined by the absence of diagnosis codes for colorectal cancer, inflammatory bowel disease or diverticular disease. Percentages are survey‐weighted estimates. Groups are not mutually exclusive. ICD‐10‐CM, International Classification of Diseases, 10th Revision, Clinical Modification.

## Data Availability

The data that support the findings of this study are available from Healthcare Cost and Utilization Project (HCUP). Restrictions apply to the availability of these data, which were used under license for this study. Data are available from https://hcup‐us.ahrq.gov/tech_assist/centdist.jsp with the permission of Healthcare Cost and Utilization Project (HCUP).
