# Supplementary material for: Outcome‐specific risk factors for dehydration and acute kidney injury readmission after ileostomy: A US National Cohort Study, 2016–2022
Source: Colorectal Dis. 2026 Oct 26;28(11):e70656. doi: 10.1111/codi.70656 (PMC13702829; doi:10.1111/codi.70656)
Supplement: Supplementary file 1 [file CODI-28-0-s001.docx]

**Supplemental Table S1. Risk factors across readmission outcomes**

| Risk Factor | 30-Day Composite, aOR (95% CI) | 30-Day Dehydration, aOR (95% CI) | 30-Day AKI, aOR (95% CI) | 60-Day Composite, aOR (95% CI) | 90-Day Composite, aOR (95% CI) |
| --- | --- | --- | --- | --- | --- |
| Discharged home (vs Facility) | 1.00 (0.96–1.05) | 1.25 (1.19–1.31)* | 0.92 (0.88–0.96)* | 0.87 (0.84–0.90)* | 0.82 (0.79–0.85)* |
| Colorectal Cancer (vs No) | 0.99 (0.96–1.03) | 1.03 (0.98–1.07) | 1.02 (0.97–1.06) | 1.01 (0.98–1.04) | 1.03 (1.00–1.07)* |
| Diverticular Disease (vs No) | 0.98 (0.94–1.02) | 1.03 (0.99–1.08) | 1.02 (0.97–1.07) | 0.98 (0.94–1.02) | 0.97 (0.94–1.01) |
| Inflammatory Bowel Disease (vs No) | 0.92 (0.87–0.96)* | 0.99 (0.94–1.05) | 0.74 (0.70–0.78)* | 0.89 (0.85–0.93)* | 0.89 (0.86–0.93)* |
| Elective admission (vs No) | 1.09 (1.05–1.13)* | 1.12 (1.08–1.17)* | 1.07 (1.03–1.12)* | 1.05 (1.01–1.09)* | 1.04 (1.00–1.07)* |
| Congestive heart failure (vs No) | 1.07 (1.01–1.13)* | 0.97 (0.90–1.04) | 1.06 (1.00–1.14) | 1.09 (1.03–1.15)* | 1.11 (1.06–1.17)* |
| Diabetes (complicated) (vs No) | 1.46 (1.39–1.53)* | 1.45 (1.37–1.53)* | 1.51 (1.43–1.59)* | 1.46 (1.40–1.53)* | 1.47 (1.41–1.54)* |
| Fluid/Electrolyte disorder (vs No) | 1.36 (1.31–1.41)* | 1.37 (1.31–1.42)* | 1.36 (1.31–1.42)* | 1.39 (1.35–1.44)* | 1.45 (1.40–1.50)* |
| Hypertension (complicated) (vs No) | 1.18 (1.09–1.26)* | 1.18 (1.09–1.28)* | 1.25 (1.16–1.36)* | 1.19 (1.11–1.27)* | 1.20 (1.13–1.28)* |
| Renal failure (vs No) | 1.28 (1.20–1.37)* | 1.07 (0.99–1.16) | 1.37 (1.27–1.48)* | 1.35 (1.27–1.44)* | 1.39 (1.31–1.48)* |
| Sex (vs Male) | 1.08 (1.05–1.12)* | 1.14 (1.10–1.18)* | 1.03 (0.99–1.07) | 1.12 (1.09–1.15)* | 1.12 (1.09–1.15)* |
| Hospital Bed Size (Large) | Ref | Ref | Ref | Ref | Ref |
| Hospital Bed Size (Medium) | 1.05 (1.01–1.09)* | 1.07 (1.02–1.12)* | 1.06 (1.01–1.11)* | 1.04 (1.00–1.08)* | 1.02 (0.98–1.05) |
| Hospital Bed Size (Small) | 1.03 (0.98–1.08) | 1.06 (1.00–1.12)* | 1.05 (0.99–1.10) | 1.02 (0.97–1.06) | 1.00 (0.96–1.04) |
| Urban/Rural teaching (Urban teaching) | Ref | Ref | Ref | Ref | Ref |
| Urban/Rural teaching (Rural) | 0.97 (0.90–1.05) | 1.02 (0.93–1.11) | 0.96 (0.88–1.04) | 0.94 (0.87–1.01) | 0.90 (0.83–0.96)* |
| Urban/Rural teaching (Urban non-teaching) | 1.05 (1.01–1.09)* | 1.09 (1.04–1.15)* | 1.05 (1.01–1.11)* | 1.03 (0.99–1.07) | 1.02 (0.99–1.06) |
| Extended Length of Stay (>7 days) (vs No) | 1.48 (1.42–1.53)* | 1.42 (1.36–1.48)* | 1.50 (1.44–1.57)* | 1.51 (1.46–1.56)* | 1.50 (1.46–1.56)* |
| Household Income (Q4 (Highest)) | Ref | Ref | Ref | Ref | Ref |
| Household Income (Q1 (Lowest)) | 1.17 (1.12–1.23)* | 1.12 (1.07–1.18)* | 1.26 (1.20–1.33)* | 1.17 (1.12–1.22)* | 1.17 (1.13–1.22)* |
| Household Income (Q2) | 1.13 (1.08–1.18)* | 1.10 (1.05–1.16)* | 1.19 (1.13–1.26)* | 1.12 (1.07–1.17)* | 1.11 (1.07–1.16)* |
| Household Income (Q3) | 1.03 (0.99–1.08) | 1.02 (0.97–1.08) | 1.07 (1.02–1.13)* | 1.03 (0.99–1.07) | 1.03 (0.99–1.07) |
| Age ≥ 65 years (vs No) | 1.46 (1.41–1.51)* | 1.46 (1.40–1.51)* | 1.66 (1.59–1.72)* | 1.48 (1.43–1.52)* | 1.47 (1.42–1.51)* |
| Ileostomy with concurrent colorectal resection (vs without) | 0.89 (0.86–0.92)* | 0.89 (0.86–0.93)* | 0.88 (0.85–0.91)* | 0.87 (0.85–0.89)* | 0.87 (0.84–0.89)* |
