# Supplementary material for: Outcome‐specific risk factors for dehydration and acute kidney injury readmission after ileostomy: A US National Cohort Study, 2016–2022
Source: Colorectal Dis. 2026 Oct 26;28(11):e70656. doi: 10.1111/codi.70656 (PMC13702829; doi:10.1111/codi.70656)
Supplement: Supplementary file 2 [file CODI-28-0-s002.docx]

**Supplemental Table S3. Risk factors for 30-day readmission for dehydration or AKI by outcome diagnosis position**

| Risk Factor | Any DX Position, aOR (95% CI) | Principal DX Only, aOR (95% CI) |
| --- | --- | --- |
| Discharged home (vs Facility) | 1.00 (0.96–1.05) | 1.33 (1.25–1.42)* |
| Colorectal Cancer (vs No) | 0.99 (0.96–1.03) | 1.12 (1.06–1.19)* |
| Diverticular Disease (vs No) | 0.98 (0.94–1.02) | 1.23 (1.15–1.31)* |
| Inflammatory Bowel Disease (vs No) | 0.92 (0.87–0.96)* | 0.83 (0.77–0.89)* |
| Elective admission (vs No) | 1.09 (1.05–1.13)* | 1.17 (1.10–1.23)* |
| Congestive heart failure (vs No) | 1.07 (1.01–1.13)* | 1.00 (0.91–1.10) |
| Diabetes (complicated) (vs No) | 1.46 (1.39–1.53)* | 1.45 (1.34–1.56)* |
| Fluid/Electrolyte disorder (vs No) | 1.36 (1.31–1.41)* | 1.20 (1.13–1.27)* |
| Hypertension (complicated) (vs No) | 1.18 (1.09–1.26)* | 1.21 (1.08–1.36)* |
| Renal failure (vs No) | 1.28 (1.20–1.37)* | 1.26 (1.14–1.40)* |
| Sex (vs Male) | 1.08 (1.05–1.12)* | 1.16 (1.11–1.22)* |
| Hospital Bed Size (Large) | Ref | Ref |
| Hospital Bed Size (Medium) | 1.05 (1.01–1.09)* | 1.05 (0.99–1.11) |
| Hospital Bed Size (Small) | 1.03 (0.98–1.08) | 1.01 (0.94–1.09) |
| Urban/Rural teaching (Urban teaching) | Ref | Ref |
| Urban/Rural teaching (Urban non-teaching) | 1.05 (1.01–1.09)* | 1.04 (0.97–1.11) |
| Urban/Rural teaching (Rural) | 0.97 (0.90–1.05) | 1.03 (0.91–1.15) |
| Extended Length of Stay (>7 days) (vs No) | 1.48 (1.42–1.53)* | 1.49 (1.40–1.58)* |
| Household Income (Q4 (Highest)) | Ref | Ref |
| Household Income (Q1 (Lowest)) | 1.17 (1.12–1.23)* | 1.28 (1.19–1.38)* |
| Household Income (Q2) | 1.13 (1.08–1.18)* | 1.22 (1.14–1.31)* |
| Household Income (Q3) | 1.03 (0.99–1.08) | 1.12 (1.05–1.21)* |
| Age ≥ 65 years (vs No) | 1.46 (1.41–1.51)* | 1.84 (1.75–1.95)* |
| Ileostomy with concurrent colorectal resection (vs without) | 0.89 (0.86–0.92)* | 0.89 (0.84–0.93)* |
