# Supplementary material for: Outcome‐specific risk factors for dehydration and acute kidney injury readmission after ileostomy: A US National Cohort Study, 2016–2022
Source: Colorectal Dis. 2026 Oct 26;28(11):e70656. doi: 10.1111/codi.70656 (PMC13702829; doi:10.1111/codi.70656)
Supplement: Supplementary file 3 [file CODI-28-0-s003.docx]

**Supplemental Table S6. Diagnoses documented among patients in the Other indication category**

| Diagnosis group | ICD-10-CM code(s) | % |
| --- | --- | --- |
| Peritonitis or sepsis | K65, A40–A41, R65.2 | 47.6 |
| Obstruction or ileus | K56 | 39.6 |
| Vascular disorder of intestine | K55 | 24.2 |
| Intestinal abscess, perforation, or fistula | K63.0–K63.2 | 23.3 |
| Other malignant neoplasm | C00–C99, excluding C18–C21 | 20.1 |
| Hernia | K40–K46 | 14.8 |
| Other functional intestinal disorder | K59 | 12.7 |
| Infectious or other colitis | A04, K52 | 9.8 |
| Benign colorectal neoplasm or polyp | D12, K63.5 | 4.8 |
| Enterostomy complication | K94.1 | 4.4 |
| Bowel trauma | S36.4–S36.5 | 3.8 |
| Appendicitis | K35–K37 | 2.5 |
| Congenital intestinal disorder | Q42–Q43 | 2.0 |
| At least one listed diagnosis group |  | 91.1 |
