# Supplementary material for: Outcome‐specific risk factors for dehydration and acute kidney injury readmission after ileostomy: A US National Cohort Study, 2016–2022
Source: Colorectal Dis. 2026 Oct 26;28(11):e70656. doi: 10.1111/codi.70656 (PMC13702829; doi:10.1111/codi.70656)
Supplement: Supplementary file 4 [file CODI-28-0-s004.docx]

**Supplemental Table S4. In-hospital mortality during index admission, stratified by indication and admission type.**

| Stratum | n | Deaths | Mortality, % |
| --- | --- | --- | --- |
| *Overall* |  |  |  |
| All patients | 189,829 | 13,679 | 7.1 |
| *By indication* |  |  |  |
| Cancer | 59,258 | 2,079 | 3.5 |
| IBD | 30,573 | 814 | 2.6 |
| Diverticulitis | 27,398 | 1,329 | 4.8 |
| Other | 72,600 | 9,457 | 12.8 |
| *By admission type* |  |  |  |
| Elective | 91,583 | 1,517 | 1.7 |
| Emergent | 98,246 | 12,162 | 12.2 |
| *Indication × admission type* |  |  |  |
| Cancer / Elective | 40,134 | 423 | 1.1 |
| Cancer / Emergent | 19,124 | 1,656 | 8.6 |
| IBD / Elective | 16,439 | 105 | 0.7 |
| IBD / Emergent | 14,134 | 709 | 4.9 |
| Diverticulitis / Elective | 11,249 | 125 | 1.1 |
| Diverticulitis / Emergent | 16,149 | 1,204 | 7.4 |
| Other / Elective | 23,761 | 864 | 3.7 |
| Other / Emergent | 48,839 | 8,593 | 17.3 |
