# Supplementary material for: Outcome‐specific risk factors for dehydration and acute kidney injury readmission after ileostomy: A US National Cohort Study, 2016–2022
Source: Colorectal Dis. 2026 Oct 26;28(11):e70656. doi: 10.1111/codi.70656 (PMC13702829; doi:10.1111/codi.70656)
Supplement: Supplementary file 5 [file CODI-28-0-s006.docx]

**Supplemental Table S2. Procedure and diagnosis codes for cohort identification, outcome ascertainment, and postoperative complications**

| Code | Description |
| --- | --- |
| *Ileostomy Procedure (ICD-10-PCS)* |  |
| 0D1B074 | Bypass ileum to cutaneous with autologous tissue substitute, open approach |
| 0D1B0J4 | Bypass ileum to cutaneous with synthetic substitute, open approach |
| 0D1B0Z4 | Bypass ileum to cutaneous, open approach |
| 0D1B4* | Bypass ileum to cutaneous, percutaneous endoscopic approach (all variants) |
| *Dehydration Diagnosis (ICD-10-CM)* |  |
| E86.0 | Dehydration |
| E86.1 | Hypovolemia |
| E86.9 | Volume depletion, unspecified |
| *Acute Kidney Injury (ICD-10-CM)* |  |
| N17.0 | Acute kidney failure with tubular necrosis |
| N17.1 | Acute kidney failure with acute cortical necrosis |
| N17.2 | Acute kidney failure with medullary necrosis |
| N17.8 | Other acute kidney failure |
| N17.9 | Acute kidney failure, unspecified |
| *Concurrent Colorectal Resection (ICD-10-PCS)* |  |
| 0DTE* | Resection of large intestine |
| 0DTF* | Resection of right large intestine |
| 0DTG* | Resection of left large intestine |
| 0DTH* | Resection of cecum |
| 0DTK* | Resection of ascending colon |
| 0DTL* | Resection of transverse colon |
| 0DTM* | Resection of descending colon |
| 0DTN* | Resection of sigmoid colon |
| 0DTP* | Resection of rectum |
| 0DQP* | Repair of rectum |
| *Colorectal Cancer (ICD-10-CM)* |  |
| C18* | Malignant neoplasm of colon |
| C19 | Malignant neoplasm of rectosigmoid junction |
| C20 | Malignant neoplasm of rectum |
| C21* | Malignant neoplasm of anus and anal canal |
| *Inflammatory Bowel Disease (ICD-10-CM)* |  |
| K50* | Crohn’s disease |
| K51* | Ulcerative colitis |
| *Diverticular Disease (ICD-10-CM)* |  |
| K57* | Diverticular disease of intestine |
| *Postoperative Complication Set (ICD-10-CM)* |  |
| N17.0–N17.2, N17.8–N17.9 | Acute kidney injury |
| E87.0–E87.6, E87.8, E83.3–E83.5 | Electrolyte imbalance |
| A40.0, A40.1, A40.3, A40.8–A40.9, A41.01–A41.02, A41.1–A41.4, A41.50–A41.53, A41.59, A41.81, A41.89, A41.9, R65.20–R65.21, T81.44 | Sepsis or septic shock |
| K56.0, K56.50–K56.52, K56.7, K91.30–K91.32 | Ileus or intestinal obstruction |
| T81.30–T81.33, T81.40–T81.44, T81.49, L03.0–L03.3, L03.8–L03.9 | Surgical site infection |
| I82.40*, I82.41*, I82.42*, I82.43*, I82.44*, I82.49*, I82.4Z*, I82.5Z*, I82.60* | Deep vein thrombosis |
| I26* | Pulmonary embolism |
| T81.30–T81.33 | Wound dehiscence |
| J80, J95.00–J95.04, J95.09, J95.811–J95.812, J95.821–J95.822, J96.00–J96.02, J96.10–J96.12, J96.20–J96.22, J96.90–J96.92, J69.0, R09.02, Z99.12, J18* | Respiratory complications |
