# Supplementary material for: Outcome‐specific risk factors for dehydration and acute kidney injury readmission after ileostomy: A US National Cohort Study, 2016–2022
Source: Colorectal Dis. 2026 Oct 26;28(11):e70656. doi: 10.1111/codi.70656 (PMC13702829; doi:10.1111/codi.70656)
Supplement: Supplementary file 6 [file CODI-28-0-s007.docx]

**Supplemental Table S5. Annual 30-day readmission rates for dehydration or acute kidney injury after ileostomy creation**

| Year | Rate, % | 95% CI, % |
| --- | --- | --- |
| 2016 | 13.2 | 12.6–13.7 |
| 2017 | 13.5 | 12.9–14.0 |
| 2018 | 14.1 | 13.6–14.6 |
| 2019 | 14.0 | 13.5–14.5 |
| 2020 | 13.2 | 12.7–13.8 |
| 2021 | 13.3 | 12.8–13.8 |
| 2022 | 13.4 | 12.9–13.9 |
